# Long-term survival of stage 4 gallbladder cancer after extended radical surgery plus limited chemotherapy: a case report

**DOI:** 10.1093/jscr/rjaf010

**Published:** 2025-01-20

**Authors:** Zhengbin Huang, Jian Sun, Changsong Li, Sheng Chen, Tian Jin, Zhengqi Wu

**Affiliations:** Department of General Surgery, Hanchuan People’s Hospital, 1 Renmin Avenue, Hanchuan, Hubei 431600, China; Department of General Surgery, Hanchuan People’s Hospital, 1 Renmin Avenue, Hanchuan, Hubei 431600, China; Department of Radiology, Hanchuan People’s Hospital, 1 Renmin Avenue, Hanchuan, Hubei 431600, China; Department of General Surgery, Hanchuan People’s Hospital, 1 Renmin Avenue, Hanchuan, Hubei 431600, China; Department of Pathology, Hanchuan People’s Hospital, 1 Renmin Avenue, Hanchuan, Hubei 431600, China; Department of Medicine, Winchester Medical Center, 1840 Amherst Street, Winchester, VA 22601, United States

**Keywords:** gallbladder cancer, long-term survival, extended radical surgery, chemotherapy

## Abstract

Gallbladder cancers (GBC) are insidious, malignant, and associated with poor prognosis, with a 5-year survival rate of 5%. Long-term survival in advanced GBC is rare. Here, we report a case of a 45-year-old female who presented with intermittent right upper quadrant pain for 1 month. A gallbladder mass and two liver masses were identified on a computed tomography (CT) scan of the abdomen and pelvis with intravenous contrast, which was highly suspicious for GBC. The patient underwent extended radical surgery, and a low to moderately differentiated gallbladder adenocarcinoma was diagnosed through pathology. Postoperatively, the patient received chemotherapy with gemcitabine and cisplatin but only tolerated one cycle. The patient has been disease-free for over 7 years, representing an unusually long survival.

## Introduction

Gallbladder cancer (GBC) is the most common malignant tumor in the biliary system. Early GBC does not present specific symptoms, and at the time of diagnosis, many patients already exhibit local invasion and metastasis to lymph nodes (LN) or distant organs. This leads to a poor prognosis, with a 5-year survival rate of ~5% [1]. Long-term survival in GBC is very rare and often requires repeated surgery combined with chemotherapy [2–5]. Here, we report a case of stage 4 GBC with over 7 years of survival following extended radical surgery and brief chemotherapy.

## Case report

**Figure 1:**
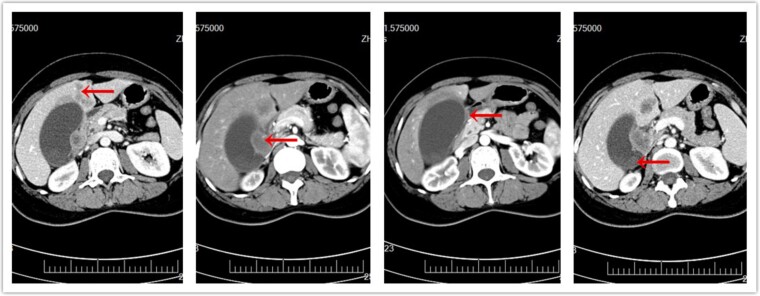
CT of abdomen prior to surgery. (a) Metastatic liver masses. (b) Enlarged lymph nodes. (c) Invasion of duodenum. (d) Original gallbladder tumor.

A 45-year-old female presented in July 2017 with intermittent right upper quadrant pain for 1 month. A computed tomography (CT) of the abdomen and pelvis with intravenous contrast revealed gallstones, a gallbladder mass, two liver masses in the left lobe, and portal lymphadenopathy (Fig. 1). The preliminary diagnosis was stage 4 GBC, for which the prognosis is poor and surgical treatment is usually not recommended. However, the patient strongly requested surgery hoping for a better outcome. We felt that the only chance for a cure in this young patient would be radical surgery to achieve an R0 resection. This complex surgery carried a high risk of complications. The patient understood the risks and chose to proceed with the surgery after signing informed consent.

During open surgery, the following findings were noted: a gallbladder fundus mass measuring 3 × 3 cm, two masses in the left lobe of the liver (the larger mass measuring 3 × 2 cm), several hard and enlarged portal lymph nodes, and adhesions of the gallbladder to the duodenum and colon. We performed an extended radical surgery in two steps [6]:

1. **En bloc resection:** liver segment IVB and V (with gallbladder), extrahepatic bile duct, intra-hepatoduodenal ligament lymph nodes and fat tissue, distal stomach, partial duodenum, and hepatic flexure colon.
2. **Reconstructions:** The ileum, 20 cm distal to the Treitz ligament, was severed and elevated to perform an anastomosis with the common hepatic duct (Roux-en-Y anastomosis), stomach, and proximal ileum. Then, we performed a colon side-to-side anastomosis.

Intraoperative frozen section analysis of the 13a lymph node and the distal common bile duct incision margin was negative for cancer cells. The intraoperative bleeding was ~1000 ml. During the operation, the patient received 5 units of red blood cells, 400 ml of plasma, and 8 units of cryoprecipitate.

Postoperative pathology demonstrated the following:

1. A 2.5 × 2.5 × 2 cm mass invading the entire depth of the gallbladder, the duodenal serosa, and multiple nerves, with positive cancer clots in the vessels and multiple metastases to the liver. One of 34 surrounding gallbladder lymph nodes was positive for metastasis.
2. No cancer cells in the incision margins of the distal common bile duct, proximal common hepatic duct, stomach, duodenum, or colon.
3. No cancer cells in the greater omentum.

The pathological diagnosis was low- to moderate-differentiation gallbladder adenocarcinoma.

**Figure 2:**
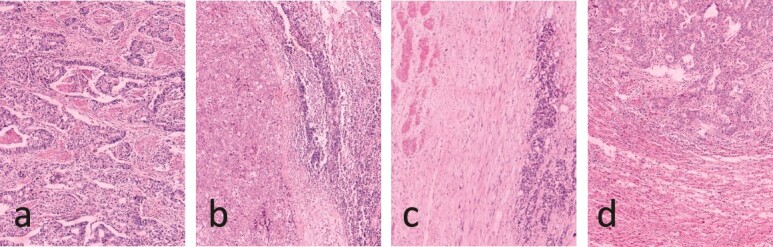
Pathology results. (a) Low to moderate differentiation gallbladder adenocarcinoma. (b) Lymphatic metastasis. (c) Duodenum wall invasion. (d) Hepatic metastasis.

Immunohistochemistry revealed CK8/18(+), CK7(+), CK20(+), p53(+), EGFR(+), CD56(+), and Ki-67(+, 80%) (Fig. 2). The final diagnosis was cT3N1M1 GBC according to the American Joint Committee on Cancer 8th edition.

Subsequently, the patient received one cycle of chemotherapy with gemcitabine and cisplatin. Further chemotherapy was not tolerated due to severe nausea and vomiting. The patient had an uneventful hospitalization without complications. At yearly follow-ups, the patient has remained disease-free for over 7 years.

## Discussion

GBC has a mean survival of 6 months and an overall 5-year survival rate of 5%. Due to its insidious onset, early diagnosis of GBC is uncommon. GBC is often identified through CT or magnetic resonance imaging (MRI) as a late-stage disease. A biopsy of the tumor is not mandatory before GBC surgery. If required, endoscopic ultrasound-guided fine needle aspiration can be utilized for tissue diagnosis. Tumor markers such as CA 19-9, CA 125, CA 242, CA 15-3, and CEA are helpful in diagnosing GBC and detecting its recurrence [7, 8].

Early-stage GBC remains curable through surgery. Tis and T1a cases require only cholecystectomy. For T1b GBC, there is controversy: some authors believe cholecystectomy is sufficient, while others recommend combined radical surgery with LN dissection. For T2 and T3 GBC, resection of segments IVB and V along with local LN dissection is suggested. In advanced GBC, extended radical surgery achieving R0 resection combined with adjuvant therapy may significantly prolong survival. Both laparoscopic and robotic surgeries are safe and effective for GBC. Intraoperative biopsy of group 13a lymph nodes should guide the extent of LN dissection. If group 13a lymph nodes are negative, only N1 and N2 dissections are required. If positive, dissection should extend to N3. Surgery is not recommended if group 16 lymph nodes are positive [9–11].

The first-line chemotherapy regimen consists of cisplatin and gemcitabine, often combined with immune checkpoint inhibitors and targeted therapy. Second-line therapies include folinic acid, 5-fluorouracil, oxaliplatin or irinotecan with 5-fluorouracil [12]. Neoadjuvant therapy can benefit patients with locally advanced, non-resectable cancer, and allowing some to undergo surgery. Radiation therapy plays a limited role but may improve prognosis in locally invasive and unresectable GBC [13].

Combining the PD-L1 monoclonal antibody Durvalumab with standard cisplatin-gemcitabine therapy has shown potential to downstage initially unresectable or metastatic GBC, making curative conversion surgery feasible. This combination may improve median overall survival and could become the new first-line treatment for advanced GBC [14].

## Conclusion

GBC is typically asymptomatic until it reaches a later stage, resulting in a poor prognosis. Surgical resection is the only potential cure. Our patient presented with late-stage GBC, evidenced by multiple liver metastases, gallbladder serosal invasion, duodenal serosa involvement, and lymphatic metastasis. We performed an extended radical surgery with negative margins in all involved organs, achieving an R0 resection. This R0 resection was likely the primary factor contributing to the patient’s long-term survival, while limited postoperative chemotherapy played a lesser role. Advances in immunotherapies and targeted therapies offer new hope for multi-modality treatment approaches in GBC, making curative surgery and long-term survival increasingly feasible.

## Data Availability

Not applicable.
